# Percutaneous Revascularization in End-Stage Kidney Disease (ESKD) Patients With Non-culprit Coronary Stenosis: A Strategy Beyond Fractional Flow Reserve

**DOI:** 10.7759/cureus.72271

**Published:** 2024-10-24

**Authors:** Alexandru Burlacu, Adrian Covic

**Affiliations:** 1 Cardiovascular Diseases, Grigore T. Popa University of Medicine and Pharmacy, Iasi, ROU; 2 Cardiology, Institute of Cardiovascular Diseases "Prof. Dr. George I.M. Georgescu", Iasi, ROU; 3 Nephrology, Grigore T. Popa University of Medicine and Pharmacy, Iasi, ROU

**Keywords:** acute myocardial infarction, chronic kidney disease, coronary hemodynamics, fractional flow reserve, myocardial revascularization

## Abstract

Complete myocardial revascularization, targeting both culprit and non-culprit coronary stenoses, is recommended by current guidelines in acute myocardial infarction (AMI) management, either during the index percutaneous coronary intervention (PCI) procedure or within 45 days, depending on the clinical context. However, in patients with chronic kidney disease (CKD), particularly end-stage kidney disease (ESKD), fractional flow reserve (FFR) presents unique challenges. Altered coronary physiology in CKD, such as arterial stiffness and microcirculatory dysfunction, affects FFR accuracy, complicating revascularization decisions. Recent evidence from large clinical trials has demonstrated no significant benefit of FFR-guided complete revascularization over culprit-only PCI in AMI patients. Current guidelines recommend complete revascularization but caution against sole reliance on FFR in CKD patients, suggesting alternative imaging techniques for improved risk assessment. Complete revascularization can be performed either during the index PCI procedure or staged during the same hospital admission, as data suggest better outcomes when revascularization is completed during the admission rather than delayed. Further research is needed to refine strategies for optimal outcomes in this high-risk population.

## Editorial

Complete myocardial revascularization, including both culprit and non-culprit coronary stenoses, has emerged as a pivotal strategy in the management of acute myocardial infarction (AMI) patients [1]. This approach aims to restore perfusion to the jeopardized myocardium, mitigate ischemic burden, and potentially improve clinical outcomes [1].

Challenges of fractional flow reserve (FFR) in end-stage kidney disease (ESKD)

However, advanced chronic kidney disease (CKD), particularly end-stage kidney disease (ESKD), introduces distinctive challenges regarding myocardial revascularization strategies. Notably, in the context of advanced CKD, fractional flow reserve (FFR) exhibits some peculiarities [2,3].

Impact of coronary physiology on CKD

First, due to the altered coronary physiology in CKD, FFR values may be lower than those in patients with preserved renal function [2]. Increased arterial stiffness (often present in ESKD) reduces coronary compliance and impairs vasodilatory capacity, leading to diminished FFR values. Arterial stiffness contributes to elevated resting coronary pressure and attenuated hyperemic response, further impacting FFR measurements in CKD patients. Second, microcirculatory dysfunction, a hallmark of CKD pathophysiology, can further influence FFR measurements. 

Thus, discerning between flow-limiting epicardial coronary stenosis and microcirculatory dysfunction when interpreting FFR values in ESKD patients with intermediate or debatable coronary lesions is mandatory [2]. When one encounters intermediate lesions with decreased FFR in advanced CKD patients, it is unclear whether we are dealing with truly hemodynamically significant stenosis (thus warranting revascularization) or if it is merely the result of the unique rheology of dialysis. To stent or not to stent? - That is a question for which FFR provides no solid answers.

Furthermore, ESKD patients may exhibit distinct coronary hemodynamics compared to those with earlier stages of CKD [4]. The relationship between FFR values and the risk of target vessel failure (TVF) differs significantly among CKD severity subgroups. While TVF increases with decreasing FFR across all renal function categories, ESKD patients exhibit a remarkably higher risk of TVF at every FFR value compared to those with earlier CKD stages and non-CKD patients [4]. This disparity highlights the distinctive coronary physiology and prognostic implications in ESKD patients undergoing FFR-guided myocardial revascularization [4].

Another valuable avenue for exploring coronary physiology in ESKD patients involves the use of non-invasive imaging techniques such as cardiac magnetic resonance imaging (MRI) and positron emission tomography (PET). These modalities provide critical insights into myocardial viability and ischemic burden, offering detailed tissue characterization and perfusion imaging. Cardiac MRI, in particular, can assess myocardial fibrosis and viability without exposure to ionizing radiation, making it especially useful for ESKD patients, who often require repeated imaging. In addition, PET imaging offers superior quantification of myocardial blood flow and can detect microvascular dysfunction - an issue prevalent in CKD patients - without the procedural risks associated with invasive techniques. Integrating these non-invasive modalities into the diagnostic workup could reduce the need for invasive procedures, potentially improving patient safety while providing a more holistic understanding of ischemia and coronary disease in this high-risk population.

FFR in multivessel coronary disease: current evidence

A multinational, randomized clinical trial (FULL REVASC) published in April 2024 reported comparative data on FFR-guided complete revascularization of non-culprit lesions versus culprit lesion revascularization alone in patients presenting with ST-elevation myocardial infarction (STEMI) or very high-risk non-ST-elevation myocardial infarction (NSTEMI) [5]. During 4.8 years of follow-up, there were no differences in the primary outcome incidence between FFR-guided complete revascularization and culprit-only PCI groups. Patients from FFR-guided complete revascularization had a similar risk of death from any cause or myocardial infarction or unplanned revascularization as those who received culprit-only PCI [5]. Hence, in this study, FFR-guided complete revascularization did not demonstrate superiority over culprit-only PCI in reducing adverse cardiovascular events in patients with STEMI or very-high-risk NSTEMI and multivessel coronary artery disease [5].

Diverging results from clinical trials

Opposing results were reported in previous studies addressing non-culprit stenosis management. In the DANAMI-3-PRIMULTI clinical trial, FFR-guided complete revascularization significantly reduced the risk of cardiovascular adverse events compared to treatment of the infarct-related artery only [6]. Nevertheless, the decrease in adverse events was attributed to a significant reduction in the necessity for repeat revascularization [6]. In the COMPLETE clinical trial, complete revascularization significantly reduced the risk of cardiovascular death or myocardial infarction compared to culprit-only PCI in STEMI patients with multivessel disease [1].

Guidelines/recommendations

The European Society of Cardiology guidelines on acute coronary syndromes management provide flexibility, allowing complete revascularization to be performed either during the index PCI procedure or within a 45-day window, without favoring one approach over the other. The ongoing COMPLETE 2 trial (NCT05701358) may provide further insights into the optimal timing and strategy for complete revascularization in patients with STEMI and multivessel disease, potentially influencing future guidelines. The decision to perform PCI of non-culprit stenosis should rely on the severity of angiographic findings rather than invasive functional assessments during the index procedure [7]. Similarly, the American College of Cardiology, American Heart Association, and Society for Cardiovascular Angiography and Interventions (ACC/AHA/SCAI) guideline on coronary artery revascularization recommends undergoing staged PCI of non-culprit stenosis following successful primary PCI [8].

While the current ESC and ACC guidelines advocate for complete revascularization in patients with multivessel disease, applying these recommendations in the context of ESKD requires additional considerations. As mentioned, patients with ESKD face a significantly higher risk of TVF, which complicates the decision-making process for revascularization strategies. The increased vascular calcification, arterial stiffness, and comorbid conditions prevalent in this patient population heighten procedural risks and long-term complications.

Given these challenges, a more personalized approach that considers the patient's overall risk profile, including comorbidities, life expectancy, and quality of life, may be more suitable than a standard guideline-based strategy. Tailoring revascularization strategies for ESKD patients could involve weighing the benefits of complete revascularization against the potential risks of repeat interventions, procedural complications, and limited survival benefits. Incorporating alternative imaging methods and adopting a multidisciplinary approach involving cardiology and nephrology teams could further optimize outcomes for these patients.

In addition, understanding the interplay between plaque morphology and stent technology is crucial for optimizing outcomes in patients with CAD. Advances in stent architecture, such as thinner struts and more biocompatible polymer coatings, have reduced the risk of in-stent restenosis and thrombosis. However, stent technology presents limitations in cases of complex plaque morphology or diffuse disease. Recent developments in drug-coated balloon (DCB) technology offer a promising alternative for these challenging cases. DCBs allow for localized drug delivery without leaving a permanent implant, minimizing the risks of mechanical complications like stent fracture or malapposition. Autopsy studies have provided valuable insights into how different types of plaques, particularly vulnerable ones, behave post-intervention, emphasizing the need for individualized treatment strategies.

Implications for ESKD patients

In conclusion, using FFR to determine a revascularization strategy may not benefit ESKD significantly. The new findings from the FULL REVASC trial suggest that complete revascularization based on FFR may not offer significant advantages in patients presenting with AMI. This effect could be further attenuated by ESKD, which exhibits already lower FFR values. 

Future directions and research needs

Considering these uncertainties, there is a need for further investigation using alternative risk assessment methods, including plaque (instability) analysis with intravascular imaging techniques like optical coherence tomography or thermography.

Although alternative imaging techniques such as OCT and thermography hold promise for plaque characterization and risk stratification, several limitations must be considered, particularly in the context of ESKD. First, both modalities come with significant costs, which may limit their widespread use, especially in resource-limited settings. In addition, the availability of these technologies can vary, with specialized centers being more likely to offer them, potentially restricting access for many ESKD patients. Moreover, the operator dependency of these imaging techniques introduces variability in results, as their accuracy heavily relies on the skill and experience of the interventional cardiologist. This can lead to inconsistencies in interpretation, particularly in challenging cases with diffuse or heavily calcified disease. The risks associated with invasive imaging in ESKD patients warrant careful consideration. The cumulative contrast burden during procedures could exacerbate vascular complications or further compromise residual kidney function in those not yet on dialysis. Thus, while OCT and thermography provide valuable insights, their use should be judiciously weighed against the potential procedural risks and logistical limitations.

Until these methods are validated and translated into clinical practice, the current approach of visually guided complete stenting may remain the preferred strategy, particularly in cases where hemodynamic stability is crucial, as in patients undergoing dialysis.

In addition to exploring alternative risk assessment methods, future research should also focus on the development of hybrid imaging techniques that combine both functional and anatomical assessment. For instance, merging data from modalities such as cardiac MRI, CT angiography, and PET could offer a more comprehensive understanding of both plaque burden and myocardial ischemia, particularly in the ESKD population, where traditional assessments (FFR) may be less reliable.

Identifying biomarkers that can more accurately stratify risk in this high-risk group could help in optimizing revascularization strategies and long-term management. Biomarkers related to inflammation, oxidative stress, and vascular calcification are potential candidates for further investigation.

Finally, future clinical trials should compare hybrid imaging techniques with traditional methods or evaluate the utility of novel biomarkers in guiding revascularization decisions, which could provide much-needed clarity. Such studies would ideally involve multidisciplinary collaboration between cardiologists, nephrologists, and radiologists to ensure comprehensive management of these complex patients.
